# Pandemic Stringency Measures and Hospital Admissions for Eating Disorders

**DOI:** 10.1001/jamapediatrics.2024.2044

**Published:** 2024-07-08

**Authors:** Nadia Roumeliotis, Matthew Carwana, Katia Charland, Ofélie Trudeau, Mike Benigeri, Mamadou Diop, Holly Agostino, Kate Zinszer, Isra Amsdr, Baudoin Forgeot d’Arc, Sylvana Côté, Nicole E. Basta, Patricia Fontela, Soren Gantt, Terry P. Klassen, Caroline Quach, Quynh Doan

**Affiliations:** 1 Department of Pediatrics, CHU Sainte-Justine, Montreal, Quebec, Canada; 2 Faculty of Medicine, University of Montreal, Montreal, Quebec, Canada; 3 Department of Pediatrics, University of British Columbia, Vancouver, British Columbia, Canada; 4 BC Children’s Hospital Research Institute, Vancouver, British Columbia, Canada; 5 School of Public Health, Université de Montreal, Montreal, Quebec, Canada; 6 CHU Sainte-Justine Research Center, Montreal, Quebec, Canada; 7 Institut National d’Excellence en Santé et en Services Sociaux, Montreal, Quebec, Canada; 8 Department of Pediatrics, Montreal Children’s Hospital, McGill University Health Center, Montreal, Quebec, Canada; 9 Centre for Public Health Research, Université de Montreal, Montreal, Quebec, Canada; 10 Ontario College of Art and Design University, Toronto, Ontario, Canada; 11 Department of Psychiatry, CHU Sainte-Justine, Montreal, Quebec, Canada; 12 Department of Epidemiology, Biostatistics, and Occupational Health, School of Population and Global Health, Faculty of Medicine and Health Sciences, McGill University, Montreal, Quebec, Canada; 13 Department of Microbiology, Infectious Diseases & Immunology, University of Montreal, Montreal, Quebec, Canada; 14 Children’s Hospital Research Institute of Manitoba, University of Manitoba, Winnipeg, Manitoba, Canada

## Abstract

**Importance:**

Hospitalizations for eating disorders rose dramatically during the COVID-19 pandemic. Public health restrictions, or stringency, are believed to have played a role in exacerbating eating disorders. Few studies of eating disorders during the pandemic have extended to the period when public health stringency restrictions were lifted.

**Objective:**

To assess the association between hospitalization rates for eating disorders and public health stringency during the COVID-19 pandemic and after the easing of public health restrictions.

**Design, Setting, and Participants:**

This Canadian population-based cross-sectional study was performed from April 1, 2016, to March 31, 2023, and was divided into pre–COVID-19 and COVID-19–prevalent periods. Data were provided by the Canadian Institute for Health Information and the Institut National d’Excellence en Santé et Services Sociaux for all Canadian provinces and territories. Participants included all children and adolescents aged 6 to 20 years.

**Exposure:**

The exposure was public health stringency, as measured by the Bank of Canada stringency index.

**Main Outcomes and Measures:**

The primary outcome was hospitalizations for a primary diagnosis of eating disorders (*International Statistical Classification of Diseases and Related Health Problems, Tenth Revision* code F50), stratified by region, age group, and sex. Interrupted time series analyses based on Poisson regression were used to estimate the association between the stringency index and the rate of hospitalizations for eating disorders.

**Results:**

During the study period, there were 11 289 hospitalizations for eating disorders across Canada, of which 8726 hospitalizations (77%) were for females aged 12 to 17 years. Due to low case counts in other age-sex strata, the time series analysis was limited to females within the 12- to 17-year age range. Among females aged 12 to 17 years, a 10% increase in stringency was associated with a significant increase in hospitalization rates in Quebec (adjusted rate ratio [ARR], 1.05; 95% CI, 1.01-1.07), Ontario (ARR, 1.05; 95% CI, 1.03-1.07), the Prairies (ARR, 1.08; 95% CI, 1.03-1.13), and British Columbia (ARR, 1.11; 95% CI, 1.05-1.16). The excess COVID-19–prevalent period hospitalizations were highest at the 1-year mark, with increases in all regions: Quebec (RR, 2.17), Ontario (RR, 2.44), the Prairies (RR, 2.39), and British Columbia (RR, 2.02).

**Conclusion and Relevance:**

In this cross-sectional study of hospitalizations for eating disorders across Canada, hospitalization rates for eating disorders in females aged 12 to 17 years were associated with public health measure stringency. The findings suggest that future pandemic preparedness should consider implications for youths at risk for eating disorders and their resource and support needs.

## Key Points

**Question:**

Was there an association between COVID-19 pandemic public health stringency measures and hospitalizations for eating disorders?

**Findings:**

In this Canadian population-based cross-sectional study of 8726 hospitalizations for eating disorders among female children and adolescents aged 12 to 17 years, a 10% increase in stringency was associated with significant increases in hospitalizations for eating disorders that varied according to region.

**Meaning:**

The findings suggest that future pandemic public health measures designed to mitigate direct harm from infection should consider youths at risk for eating disorders and their hospital resource and outpatient support needs.

## Teaser

This cross-sectional study investigates the association between COVID-19 public health stringency measures and hospitalization for eating disorders during the pandemic and after public health restrictions were eased.

## Introduction

Public health measures deployed to minimize hospitalizations and death from COVID-19 impacted multiple domains of child and youth well-being indirectly.^1,2,3^ In particular, an association between the pandemic period and an increase in mental health conditions, including eating disorder presentations, has been described.^4,5^

Eating disorders are a group of heterogeneous illnesses that significantly impact the psychosocial and medical health of children and adolescents. It is estimated that 5% of Canadian adolescents are affected by eating disorders.^6,7^ Eating disorders can lead to complex and life-threatening complications, with anorexia nervosa having the highest mortality rate of any psychiatric disorder. These disorders are associated with a range of adverse health outcomes, including cardiac dysrhythmias, hypotension, decreased bone mineral density, growth arrest,^8^ and a high rate of comorbid mental health disorders.^9,10^

Studies have described a rise in eating disorder hospitalizations throughout pediatric centers in the early COVID-19 pandemic,^4,11,12,13^ with many hypothesizing an association between lockdowns and exacerbations of eating disorders.^4,5,14^ Few studies, however, have isolated the association between pandemic restrictions and increased hospitalizations.^15^ Among G10 nations, Canada had the second-highest stringency, but the approach throughout the country was heterogeneous.^16,17,18^ Understanding the relationship between the stringency of pandemic public health restrictions and eating disorders and, in particular, eating disorder hospitalizations can allow better deployment of prevention and early intervention strategies. Delineating this relationship will have important implications for future pandemic preparedness. The objective of this study was to measure the association between pandemic public health stringency and the rates of hospitalizations for eating disorders among Canadian youths.

## Methods

### Study Design and Population

This Canadian population-based, repeated cross-sectional ecological study aggregated data at the regional level. The study period was from April 1, 2016, to March 31, 2023, and was divided into pre–COVID-19 and COVID-19–prevalent periods. The study population was school-aged children and adolescents aged 6 to 20 years who were eligible for provincial medical insurance from all Canadian provinces and territories. Our goal was to evaluate the association between hospitalization rates for eating disorders and COVID-19 public health stringency while accounting for prepandemic trends. The study followed the Reporting of Studies Conducted Using Observational Routinely-Collected Data (RECORD) extension of the Strengthening the Reporting of Observational Studies in Epidemiology (STROBE) reporting guideline.^19^ Research ethics board approval was provided by Centre Hospitalier Universitaire Sainte-Justine. Informed consent was waived because deidentified health administrative data were used.

### Data Sources

Data for hospitalizations from the Discharge Abstract Database (DAD) were provided by the Canadian Institute for Health Information for 9 provinces and 3 territories (all regions other than Quebec). The DAD provided complete and comprehensive data on all hospitalizations, including discharge diagnoses, and coordinated access to demographic, social, and medical administrative data, including pan-Canadian organizations, such as Census Canada. Exclusions included persons with an invalid or missing medical number, including refugees, asylum claimants, persons who left Canada for more than 6 months per year, and visitors. Individual patient-level demographic data were linked deterministically to hospital admission-level data provided by the DAD. Quebec aggregate hospitalization data were from the Maintenance et Exploitation des Données pour l’Étude de la Clientèle Hospitalière (MED-ECHO), provided by the Institut National d’Excellence en Santé et Services Sociaux, creating a complete national sample. The aggregated Quebec data were provided at the admission level, comprehensive of demographics from Census Canada for all hospitalizations for persons with a valid health card in Quebec. The sampling frame, aggregation, and available demographic variables were the same in both health administrative datasets (DAD and MED-ECHO). Patient transfers between acute care facilities were considered as 1 hospital encounter, and the lengths of stay were combined. For the incidence rate denominator, estimates of Canada’s population by region, sex, and age group were provided by Statistics Canada (estimates on July 1 of each year). In both datasets, sex is defined as sex associated with the provincial health card number, assigned at birth. Data on patient gender and race and ethnicity are not routinely collected in the Canadian health care system and, therefore, were not available in the data. To understand whether hospitalizations were new or were recurrent hospitalizations for a given patient, we included a 2-year calendar look-back period as far as April 1, 2014, to assess for mental health presentations, including eating disorder presentations.

The exposure of interest was the regional stringency of public health measures, provided by the stringency index of the Bank of Canada.^20^ The stringency index collected government policy and restrictions, measured systematically over time and across regions, following methods of the Oxford COVID-19 government response tracker.^21^ The index included 12 stringency indicators, including work or office and school closures, public event cancellations, restrictions on private gatherings, travel restrictions, stay-at-home orders, and public information campaigns, with coding that ranged from 0 to 100, with higher values indicating stricter measures (eTable 1 in Supplement 1).^16,20^ The stringency index was measured daily (eFigure 1 in Supplement 1).

### Outcomes

The primary outcome was number and rate of hospitalizations to any inpatient facility for a primary diagnosis of any type of eating disorder, identified using *International Statistical Classification of Diseases and Related Health Problems, Tenth Revision* codes F50.0 to 50.9. Although finer temporal resolutions were attempted, to provide sufficient counts, admissions were aggregated by 4-week periods and by Canadian region. For the 4-week segmented analysis, the study period was divided into the pre–COVID-19 period from April 3, 2016, to February 29, 2020, and the COVID-19–prevalent period from March 1, 2020, to March 25, 2023. March 2020 was included in the pandemic period, as restrictions began March 13, 2020, across Canada. The study period was extended to March 2023 to include a phase characterized by the removal of COVID-19 public health measures. Regions included Atlantic Canada (Newfoundland and Labrador, Nova Scotia, New Brunswick, and Prince Edward Island), Quebec, Ontario, the Prairies (Manitoba, Saskatchewan, and Alberta), British Columbia, and the Territories (Yukon, the Northwest Territories, and Nunavut) (eFigure 2 in Supplement 1).

### Variables of Interest

To match the temporal partition of the outcome, the exposure of interest was the stringency index, which was taken as the maximum daily value in each 4-week interval from March 2020 to March 2023. A lag of 12 to 20 weeks was advised by clinical experts (H.A., B.F.D.) to reflect a reasonable delay between a change in stringency and a change in hospitalizations for eating disorders. In addition to variables to account for seasonality, 3 additional variables were included as covariates in the interrupted time series (segmented) regressions. The first was an indicator for the pandemic period, taking values 0 before the pandemic and 1 afterward. The second was a linear term for the time since the start of the study period, taking consecutive values from 1 to 91, which represented the overall prepandemic trend beginning in April 2016. A third variable was included as a confounder representing the early weeks of the pandemic when health care access was (perceived as) restricted.^22,23^ This accounted for sharp decreases documented in all-cause admissions from 12 to 16 weeks after the start of the pandemic.^22,23^ This variable was included in the model as a potential confounder of the association between eating disorder admissions and stringency. While the lag for stringency was expected to be similar across regions, the duration of restricted health care access was expected to vary by region due to regional differences in COVID-19 incidence and time needed to prepare health services for the expected influx of patients with COVID-19.

### Statistical Analysis

Analyses were planned a priori to be carried out for each sex, age group, and region stratum separately. However, results are presented only for strata with sufficient cell counts. For age-sex-region strata with sufficient counts at the 4-week temporal resolution, interrupted time series analyses by segmented regressions were used to estimate the association between rate of hospitalizations for eating disorders and public health stringency.

The interrupted time series model used Poisson regression with the number of hospitalizations as the outcome and the log-transformed population size as the offset.^24^ Seasonality was modeled with harmonic pairs.^25^ To account for any unmodeled temporal correlation, we used robust SE estimation based on the Newey-West estimator.^26^ To decide between a model with a health care restriction period of 12 or 16 weeks and a stringency lag of 12 to 20 weeks, we assessed the residuals and compared the bayesian information criterion (BIC). Models with residuals exhibiting patterns of poor fit or failing the Breusch-Godfrey test were excluded from further consideration. For models passing residual diagnostics, a difference in BIC of 6 or more units was used as evidence of superior fit for the model with lower BIC. Since 2 or more candidate models could have comparable BIC, sensitivity analyses were carried out for combinations of the lag for stringency and the duration of restricted health care access.

Adjusted rate ratios and their corresponding 95% CIs were calculated for all covariates. For the stringency index, the adjusted rate ratio was interpreted for a 10% change in stringency. To assess excess hospitalizations in the COVID-19–prevalent period, we determined the ratio of the observed hospitalization rate during the COVID-19–prevalent period to the expected (counterfactual) rate had pre–COVID-19 trends continued beyond March 2020. Similarly, we computed the ratio of fitted rate vs expected (counterfactual) rate after March 2020. All measures of association were presented as relative differences. A 2-sided *P* < .05 significance level was assumed. All analyses were carried out using R, version 4.3.1 (R Project for Statistical Computing).^27^

## Results

During the study period, there were 11 289 hospitalizations for eating disorders across Canada among 6.3 million youths. Descriptive characteristics of hospitalizations for eating disorders by fiscal year, age group, and regions are provided in eTable 2 in Supplement 1 (population data are given in eTable 3 in Supplement 1). A total of 58.6% of admissions in both periods were for youths who had never previously been hospitalized with an eating disorder (eTable 2 in Supplement 1). For all other age-sex-region strata, unadjusted analyses of hospitalizations during each year are presented in eTable 4 in Supplement 1, demonstrating relative increases in eating disorder hospitalization rates in both males and females aged 6 to 17 years in all regions during the COVID-19–prevalent period compared with the prepandemic period.

Females accounted for 90.4% of all hospitalizations during our study period (9.6% males), and 8726 of all hospitalizations (77%) were of females aged 12 to 17 years. Given the low counts in all other age-sex strata, the interrupted time series analyses were restricted to females aged 12 to 17 years, with the descriptive characteristics of eating disorder hospitalizations for this stratum presented by fiscal year in Table 1.

**Table 1.** Characteristics of Hospitalizations for Eating Disorders Among Females Aged 12 to 17 Years by Fiscal Year^a^

Characteristic	Hospitalizations, No. (%) (N = 8726)						
	2016-2017	2017-2018	2018-2019	2019-2020	2020-2021	2021-2022	2022-2023
Total, No.	908	885	958	979	1653	1891	1452
Area^b^							
Urban	784 (87.3)	766 (87.5)	831 (88.3)	846 (88.1)	1456 (89.8)	1640 (87.9)	1256 (88.0)
Rural	114 (12.7)	109 (12.5)	110 (11.7)	114 (11.9)	166 (10.2)	225 (12.1)	172 (12.0)
Regions^c^							
Atlantic	39 (4.3)	64 (7.2)	50 (5.2)	61 (6.2)	81 (4.9)	82 (4.3)	51 (3.5)
Quebec	256 (28.2)	224 (25.3)	258 (26.9)	244 (24.9)	404 (24.4)	509 (26.9)	409 (28.2)
Ontario	400 (44.1)	389 (44.0)	412 (43.0)	444 (45.4)	779 (47.1)	886 (46.9)	689 (47.4)
Prairies	90 (9.9)	81 (9.2)	92 (9.6)	85 (8.7)	162 (9.8)	200 (10.6)	144 (9.9)
British Columbia	121 (13.3)	127 (14.3)	143 (14.9)	145 (14.8)	223 (13.5)	206 (10.9)	155 (10.7)
Mental health history							
Emergency department visit	159 (17.5)	169 (19.1)	160 (16.7)	190 (19.4)	315 (19.1)	406 (21.5)	315 (21.7)
Hospitalization	313 (34.5)	285 (32.2)	363 (37.9)	360 (36.8)	463 (28.0)	565 (29.9)	503 (34.6)
None	436 (48.0)	431 (48.7)	435 (45.4)	429 (43.8)	875 (52.9)	920 (48.7)	634 (43.7)
Eating disorder in past 2 y							
Emergency department visit	95 (10.5)	102 (11.5)	112 (11.7)	104 (10.6)	209 (12.6)	293 (15.5)	214 (14.7)
Hospitalization	296 (32.6)	254 (28.7)	325 (33.9)	321 (32.8)	430 (26.0)	507 (26.8)	461 (31.7)
None	517 (56.9)	529 (59.8)	521 (54.4)	554 (56.6)	1014 (61.3)	1091 (57.7)	777 (53.5)
Pediatric center^d^							
Yes	542 (59.7)	530 (59.9)	582 (60.8)	627 (64.0)	1063 (64.3)	1081 (57.2)	824 (56.7)
No	366 (40.3)	355 (40.1)	376 (39.2)	352 (36.0)	590 (35.7)	810 (42.8)	628 (43.3)
Length of stay, median (IQR), d^e^	16 (8-35)	16 (9-31)	15 (7-30)	14 (7-27)	14 (7-24)	13 (7-25)	12 (6-21)
Transfer to another health care facility during admission	80 (8.8)	84 (9.5)	90 (9.4)	71 (7.3)	141 (8.5)	144 (7.6)	123 (8.5)

^a^ The fiscal year in Canada is from April 1 to March 31.

^b^ Data were missing for 137 hospitalizations (1.6%).

^c^ Numbers not reported for Territories as cell counts were less than 5 (21 total hospitalizations [0.2%]).

^d^ Admission to a pediatric center was defined as a center having a tertiary care pediatric intensive care unit.

^e^ For patients transferred between 2 facilities, the length of stay for both facilities was combined.

**Figure 1.**
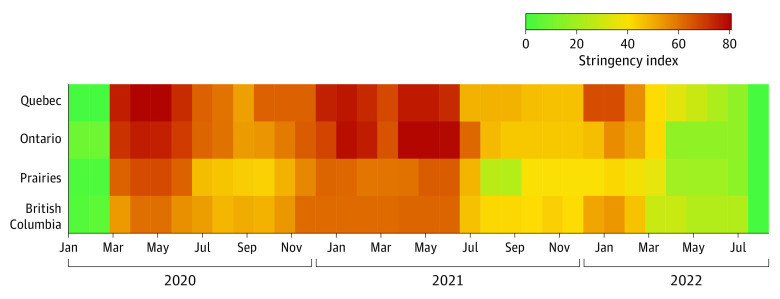
Regional Stringency Index by 4-Week Intervals Between January 2020 and July 2022 Stringency was no longer measured after July 15, 2022. The stringency index used maximum stringency per 4-week interval provided by the Bank of Canada.

The regional stringency values by 4-week period (used in the models) are shown in Figure 1. Stringency ranged from 0 to 80 and was highest, on average, in Quebec (mean [SD], 45.24 [29.10]) followed by Ontario (mean [SD], 43.06 [29.16]), Atlantic Canada (mean [SD], 38.06 [23.80]), British Columbia (mean [SD], 37.89 [23.48]), and the Prairies (mean [SD], 35.60 [24.01]). There were insufficient 4-week counts in the Territories and Atlantic Canada to conduct the time series modeling. Consequently, separate regression models were developed only for females aged 12 to 17 years for Quebec, Ontario, the Prairies, and British Columbia, as presented in Table 2 (fitted vs expected time series results are given in eFigure 3 in Supplement 1, with the model given in eTable 5 in Supplement 1). In all provinces, the regional stringency index was significantly associated with hospitalization rate for eating disorders. A 10% rise in stringency was associated with increases in hospitalization rates in Quebec (adjusted rate ratio [ARR], 1.05; 95% CI, 1.01-1.07), Ontario (ARR, 1.05; 95% CI, 1.03-1.07), the Prairies (ARR, 1.08; 95% CI, 1.03-1.13), and British Columbia (ARR, 1.11; 95% CI, 1.05-1.16) after adjustment for other covariates (Table 2). In the sensitivity analyses in which the lags and durations of health care–restricted access stringency were varied, no more than a 0.02 absolute difference in the ARR for stringency index was observed (eTable 6 in Supplement 1).

**Table 2.** Adjusted Rate Ratios Showing the Relative Change in Eating Disorder Hospitalization Rates Associated With a 10% Change in Stringency Index, the Estimated Relative Pandemic Change, and the Relative Change During Health Care Restriction^a^

Variable	Adjusted rate ratio (95% CI)	*P* value
**Quebec**		
Prepandemic slope (time)	1.00 (1.00-1.00)	.70
Pandemic-level change	1.42 (1.11-1.81)	.005
Health care restriction	0.52 (0.43-0.63)	<.001
Stringency index	1.05 (1.01-1.07)	.001
**Ontario**		
Prepandemic slope (time)	1.00 (1.00-1.00)	.65
Pandemic-level change	1.50 (1.27-1.77)	<.001
Health care restriction	0.80 (0.70-0.92)	.002
Stringency index	1.05 (1.03-1.07)	<.001
**Prairies**		
Prepandemic slope (time)	1.00 (0.99-1.00)	.67
Pandemic-level change	1.41 (0.97-2.04)	.07
Health care restriction	0.47 (0.30-0.71)	<.001
Stringency index	1.08 (1.03-1.13)	.02
**British Columbia**		
Prepandemic slope (time)	1.00 (1.00-1.01)	.66
Pandemic-level change	0.88 (0.58-1.35)	.57
Health care restriction	0.89 (0.58-1.36)	.59
Stringency index	1.11 (1.05-1.16)	.007

^a^ Adjusted rate ratios are from multivariable Poisson regression. Health care restriction for Quebec was 16 weeks; Ontario, 16 weeks; the Prairies, 12 weeks; and British Columbia, 12 weeks. The stringency lag of 16 weeks was based on residual diagnostics and on a meaningful minimization of the bayesian information criterion. Sensitivity analysis is given in eTable 4 in Supplement 1.

**Figure 2.**
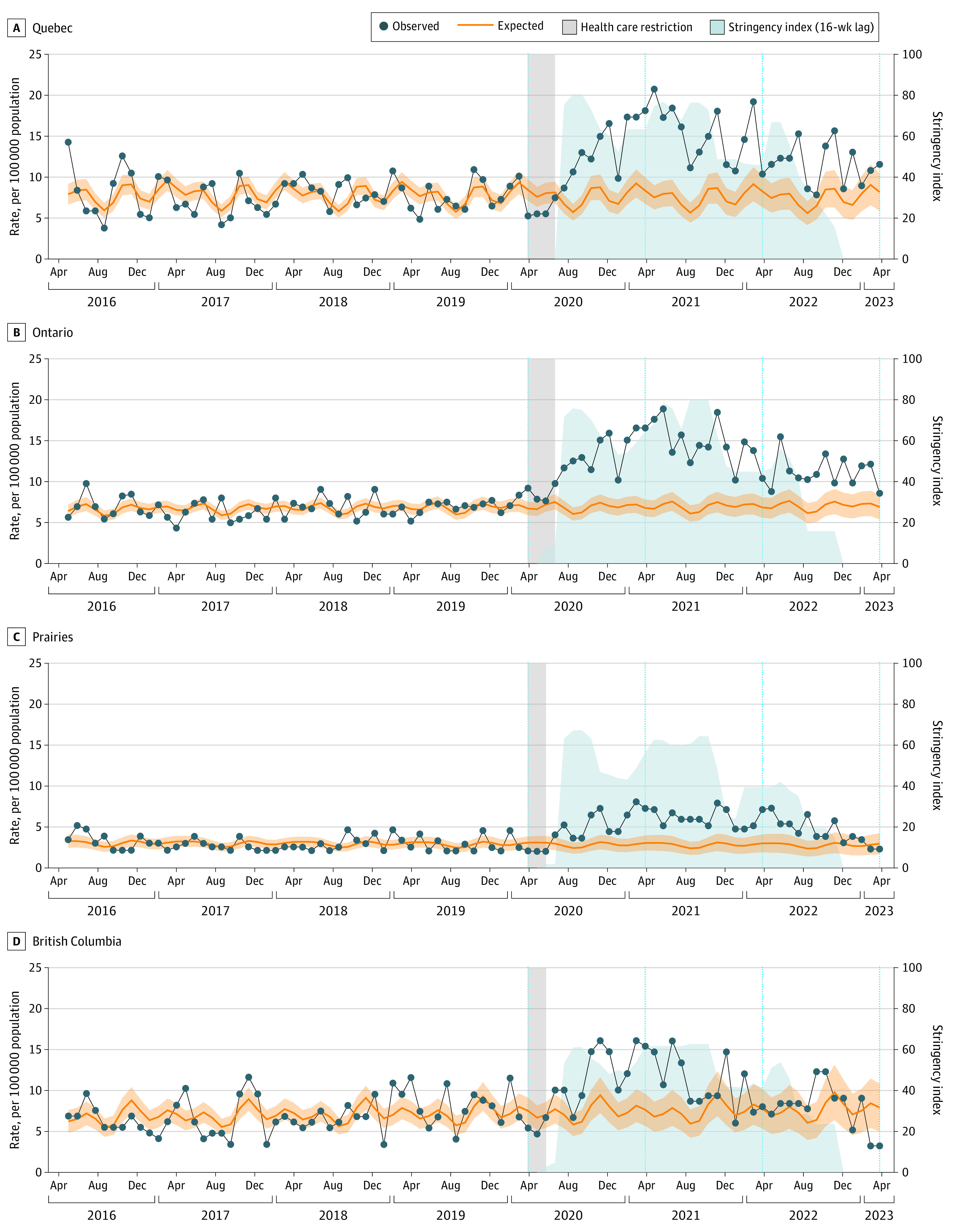
Interrupted Time Series of Eating Disorder Hospitalizations by Region and 4-Week Period From April 2016 to April 2023 Prepandemic trends were extrapolated into the COVID-19–prevalent period. “Expected” refers to counterfactual based on pre–COVID-19 pandemic trends. Expected and fitted rates are presented in eFigure 3 in Supplement 1. Shading around the expected rate line indicates 95% CIs.

For Quebec and Ontario, there was a significant pandemic-level change in eating disorder hospitalization rates from March 2020 onward, with increasing rates in Quebec (ARR, 1.42; 95% CI, 1.11-1.81) and Ontario (ARR, 1.50; 95% CI, 1.27-1.77) after adjustment for stringency and other covariates (Table 2). A time series plot with both the observed and the expected (counterfactual from prepandemic trend) eating disorder hospitalization rate series is presented in Figure 2. The change in volume of hospitalizations comparing the observed rate with the expected rate at the 1-, 2-, and 3-year mark showed that the excess hospitalizations were highest at the 1-year mark, with increases in Quebec (RR, 2.17), Ontario (RR, 2.44), the Prairies (RR, 2.39), and British Columbia (RR, 2.02) (Table 3). All regions showed decreases at the 3-year mark (Table 3).

**Table 3.** Change in Volume of Eating Disorder Hospitalizations Among Females Aged 12 to 17 Years Comparing the Expected Rate From Prepandemic Trends With the Observed Rate at the 1-, 2-, and 3-Year Mark of the COVID-19–Prevalent Period

Region, time since pandemic start^a^	Expected rate, per 100 000 population (95% CI)^b^	Observed rate, per 100 000 population	Rate ratio
**Quebec**			
1 y	8.34 (6.73-9.96)	18.12	2.17
2 y	8.26 (6.32-10.21)	10.37	1.26
3 y	8.18 (5.88-10.48)	11.56	1.41
**Ontario**			
1 y	6.77 (5.75-7.79)	16.55	2.44
2 y	6.83 (5.58-8.09)	10.39	1.52
3 y	6.89 (5.38-8.40)	8.57	1.24
**Prairies**			
1 y	3.04 (2.12-5.68)	7.27	2.39
2 y	3.00 (1.90-4.10)	7.13	2.38
3 y	2.96 (1.67-4.25)	2.30	0.78
**British Columbia**			
1 y	7.65 (5.68-9.62)	15.41	2.02
2 y	7.78 (5.36-10.20)	8.02	1.03
3 y	7.92 (5.00-10.84)	3.24	0.41

^a^ Start of pandemic indicates the 4-week period from March 1 to 28, 2020.

^b^ Expected refers to counterfactual. Expected vs fitted rates are presented in eTable 5 in Supplement 1.

## Discussion

This cross-sectional study demonstrated a significant rise in hospitalizations for eating disorders in Quebec and Ontario from March 2020 onward, predominantly among females aged 12 to 17 years. Importantly, in this age-sex strata, an increase in hospitalizations for eating disorders was associated with increased public health measure stringency across the Quebec, Ontario, Prairies, and British Columbia regions. A 10% rise in stringency was associated with a 5% increase in eating disorder hospitalization rates in Quebec and Ontario, an 8% increase in the Prairies, and an 11% increase in British Columbia after adjusting for other covariates. Atlantic Canada and the Territories had insufficient case numbers for the adjusted series regression accounting for stringency. Our findings support the hypothesis that COVID-19 public health stringency was independently associated with eating disorder hospitalizations for young females in Canada beyond a general association with the overall COVID-19–prevalent period described by previous studies.^11,12,13^ We also demonstrated that this effect was consistent across regions based on region-specific stringency measures.

To our knowledge, the direct association between public health measure stringency and hospitalization trends for eating disorders has not been previously described. This finding has important implications for future pandemic preparedness, as it suggests that the measures taken to contain direct infection may inadvertently lead to adverse health outcomes. However, defining which specific elements of public health stringency may have had the largest effect on hospitalizations and the mechanisms behind these increases is outside the scope of this study. The stringency index was constructed with multiple components of public health measures, including restrictions on gathering, masks, school closures, and restricted traveling.^28^ Stringency measures in Canada were among the highest and longest in the G10 nations.^18^ The typical child missed 51 weeks of in-person school, the second longest after the US.^17^ Emerging evidence suggests that school closures are associated with increased emergency department visits for suicidality in youths, especially among those aged 12 to 17 years^29^; it is plausible that a relationship may exist for other mental health conditions, including eating disorders. However, individual components of the stringency index, like school closure, did not exist in isolation from others. Furthermore, it is essential to maintain the perspective that the implementation of rigorous measures was aimed at mitigating COVID-19–related harm and safeguarding fragile health systems with limited resources in personnel and inpatient beds. Exploring the complexities of the association between stringency and eating disorder hospitalizations with future youth-engaged studies would be beneficial.

The relative increase in eating disorder hospitalizations during the COVID-19–prevalent period was likely multifactorial. Given that most patients with eating disorders are treated as outpatients, the lack of outpatient services during the pandemic may have led to disease progression that, when left untreated or unacknowledged, was associated with a higher likelihood of hospital admission compared with other mental health disorders given the immediate medical health risk. However, a study^13^ of a large US cohort revealed a parallel increase in hospitalizations for eating disorders despite a rise in outpatient visits, suggesting that the likely cause was not a shortage of outpatient services. Disordered eating,^30^ including binge eating,^31^ and increased compensatory exercise during periods of high stringency may also explain this increase in hospitalizations.^32^ A loss of the routine and peer relationships embedded in school and extracurricular activities and an increased amount of time spent on social media^33^ may also have been associated with exacerbated eating disorders in young females. To inform future preparedness for this at-risk population, knowledge mobilization efforts have included involving youths with lived experience in the interpretation and dissemination plan. Dissemination to general practitioners and pediatricians in Canada includes new recommendations for management of eating disorders by the Translating Emergency Knowledge for Kids team^34^ and an upcoming position statement by the Canadian Pediatric Society.

In future periods of public health restrictions, pediatricians and other health care practitioners should know that youths with eating disorders are vulnerable to relapse and require close monitoring. Physicians should find ways to stay connected with their patients for ongoing clinical assessment and psychosocial support via hospital visits or telehealth.^14^ Health care practitioners should also be screening youths for new eating disorders regardless of weight, gender, or socioeconomic status. The importance of social connectedness for youths (including support networks and parental education) should be promoted to help ensure that children, when experiencing restrictions, such as school closures, are as minimally socially isolated as possible.

### Strengths and Limitations

Our study has several unique strengths. By accessing data from both the Canadian Institute for Health Information and Institut National d’Excellence en Santé et Services Sociaux, the cohort was representative of the entire Canadian population. This study provides new knowledge using robust Canadian stringency data to assess mental health impacts of public health measures among youths. While the Canadian stringency may have been different from that in other countries, the rise in eating disorder visits was seen in many G10 countries, and the stringency index has been used in studies of physical and mental health in adults internationally,^15,16^ suggesting that these findings are possibly generalizable to other industrialized nations.

Limitations of the study are primarily related to the use of health administrative data. Identification of cases was limited to those who presented to the hospital, which likely represented a small proportion of those experiencing eating disorders; thus, our observations are only pertinent to this target population. Use of health administrative data prevented the use of gender (limited to sex at birth) and the analysis of low counts, meaning we could not present trend data for Atlantic Canada or the Territories. Furthermore, given the lack of outpatient data, we were unable to identify patients who had a new diagnosis of an eating disorder across the COVID-19–prevalent period. We attempted to mitigate this by presenting hospitalizations of patients with eating disorders who had not been to the emergency department or hospital in the previous 2 years.

## Conclusions

In this cross-sectional study, public health measures necessary to mitigate impacts of COVID-19 across Canada were significantly associated with an increase in hospitalizations for eating disorders among females aged 12 to 17 years. While causality could not be established, understanding what constraint factors in the stringency index contributed to this increase is important to inform future policy planning. The findings suggest that support through community services, psychological services, family awareness, and medical services for youths with established or newly diagnosed eating disorders will be crucial in future pandemic planning.

## Notes

Supplement 1. **eTable 1.** Policies Included in the Bank of Canada’s Stringency Index **eFigure 1.** Bank of Canada’s daily stringency index by region **eFigure 2.** Aggregation of Cases by Canadian Region **eTable 2.** Descriptive Characteristics of Hospitalizations for Eating Disorders Age 6 to 20 Years, by Fiscal Year in Canada (N = 11 289) **eTable 3.** Canadian Population Aged 6 to 20 for Each Fiscal Year From 2016 to 2022 Stratified by Region, Sex and Age Group **eTable 4.** Unadjusted Incidence Rate Ratios (IRR) for Eating Disorders, Comparing Hospitalization Rates in Pre-COVID and COVID-Prevalent Years, Stratified by Region, Sex and Age **eFigure 3.** Interrupted Time Series of Fitted vs Expected Rates of Eating Disorder Hospitalizations Among Females 12 to 17 Years of Age, by Region and 4-Week Period, From Pre-pandemic Trends **eTable 5.** Change in Volume of Eating Disorder Hospitalizations Among Females 12 to 17 Years, Comparing the Pre-pandemic Trend From the Regression Model Extrapolated Into the COVID-Prevalent Period and the Fitted Rate **eTable 6.** Sensitivity Analysis, Varying the Lag of the Stringency and the Duration of the Healthcare Restriction

Supplement 2.
Pediatric Outcomes Improvement Through Coordination of Research Networks (POPCORN) Investigators

Supplement 3.
Data Sharing Statement
