# Supplementary material for: Pandemic Stringency Measures and Hospital Admissions for Eating Disorders
Source: JAMA Pediatr. 2024 Jul 8;178(9):879–87. doi: 10.1001/jamapediatrics.2024.2044 (PMC11231909; doi:10.1001/jamapediatrics.2024.2044)
Supplement: Supplement 1. — eTable 1. Policies Included in the Bank of Canada’s Stringency Index eFigure 1. Bank of Canada’s daily stringency index by region eFigure 2. Aggregation of Cases by Canadian Region eTable 2. Descriptive Characteristics of Hospitalizations for Eating Disorders Age 6 to 20 Years, by Fiscal Year in Canada (N = 11 289) eTable 3. Canadian Population Aged 6 to 20 for Each Fiscal Year From 2016 to 2022 Stratified by Region, Sex and Age Group eTable 4. Unadjusted Incidence Rate Ratios (IRR) for Eating Disorders, Comparing Hospitalization Rates in Pre-COVID and COVID-Prevalent Years, Stratified by Region, Sex and Age eFigure 3. Interrupted Time Series of Fitted vs Expected Rates of Eating Disorder Hospitalizations Among Females 12 to 17 Years of Age, by Region and 4-Week Period, From Pre-pandemic Trends eTable 5. Change in Volume of Eating Disorder Hospitalizations Among Females 12 to 17 Years, Comparing the Pre-pandemic Trend From the Regression Model Extrapolated Into the COVID-Prevalent Period and the Fitted Rate eTable 6. Sensitivity Analysis, Varying the Lag of the Stringency and the Duration of the Healthcare Restriction [file jamapediatr-e242044-s001.pdf]

Supplementary Online Content

Roumeliotis N, Carwana M, Charland K, et al. Pandemic stringency measures and hospital admissions for eating disorders. *JAMA Pediatr*. Published online July 8, 2024. doi:10.1001/jamapediatrics.2024.2044

eTable 1. Policies Included in the Bank of Canada's Stringency Index

eFigure 1. Bank of Canada's daily stringency index by region

eFigure 2. Aggregation of Cases by Canadian Region

eTable 2. Descriptive Characteristics of Hospitalizations for Eating Disorders Age 6 to 20 Years, by Fiscal Year in Canada (N=11 289)

eTable 3. Canadian Population Aged 6 to 20 for Each Fiscal Year From 2016 to 2022 Stratified by Region, Sex and Age Group

eTable 4. Unadjusted Incidence Rate Ratios (IRR) for Eating Disorders, Comparing Hospitalization Rates in Pre-COVID and COVID-Prevalent Years, Stratified by Region, Sex and Age

eFigure 3. Interrupted Time Series of Fitted vs. Expected Rates of Eating Disorder Hospitalizations Among Females 12 to 17 Years of Age, by Region and 4-Week Period, From Pre-pandemic Trends

eTable 5. Change in Volume of Eating Disorder Hospitalizations Among Females 12 to 17 Years, Comparing the Pre-pandemic Trend From the Regression Model Extrapolated Into the COVID-Prevalent Period and the Fitted Rate

eTable 6. Sensitivity Analysis, Varying the Lag of the Stringency and the Duration of the Healthcare Restriction

This supplementary material has been provided by the authors to give readers additional information about their work.

© 2024 Roumeliotis N et al. *JAMA Pediatr*.

eTable 1. Policies included in the Bank of Canada's stringency index

Identifier
School and university closures
Workplace and office closures
Public event cancellations and restrictions
Restrictions on private gatherings
Public transport closures
Stay-at-home requirements
Restrictions on intra-provincial travel (between cities or regions within a province)
Restrictions on international travel
Restrictions on interprovincial travel (between provinces)
Enforcement mechanisms for individuals
Enforcement mechanisms for firms
Public information campaigns

Policies are measured in all 10 Canadian Provinces but not in Territories.
Model creation, data and code book can be found at link below.
Source: https://www.bankofcanada.ca/2021/02/staff-analytical-note-2021-1/#table1

eFigure 1. Bank of Canada's daily stringency index by region

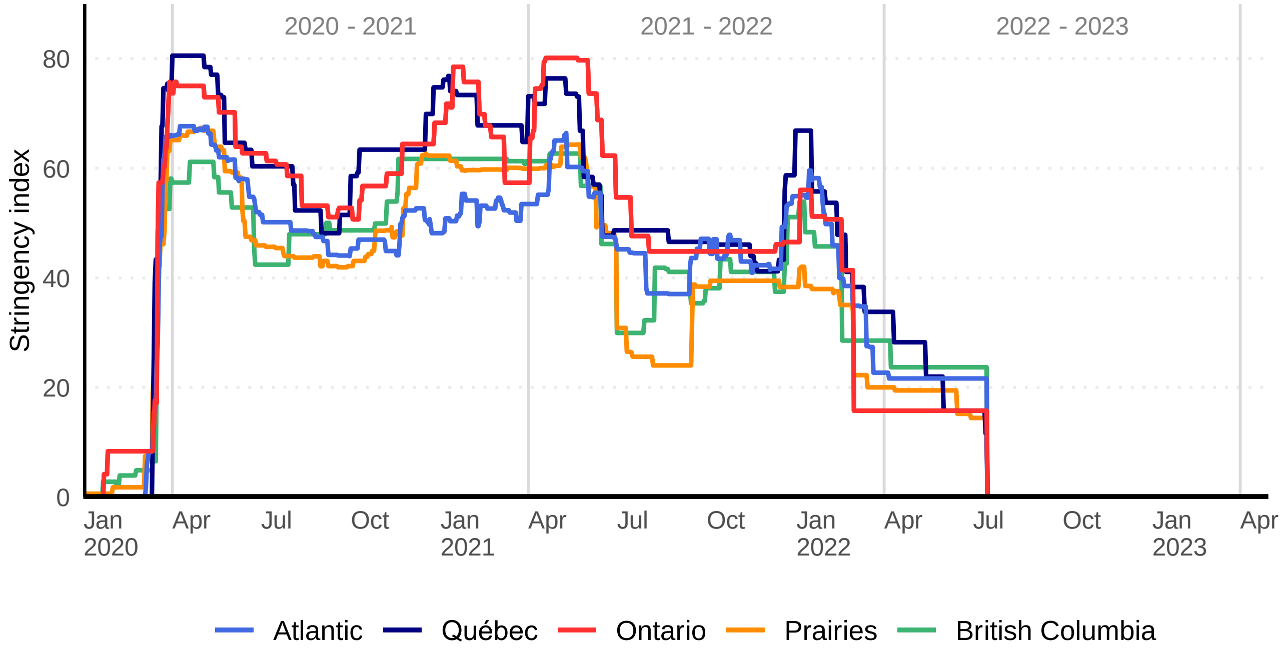

© 2024 Roumeliotis N et al. *JAMA Pediatr*.

eFigure 2. Aggregation of cases by Canadian region.

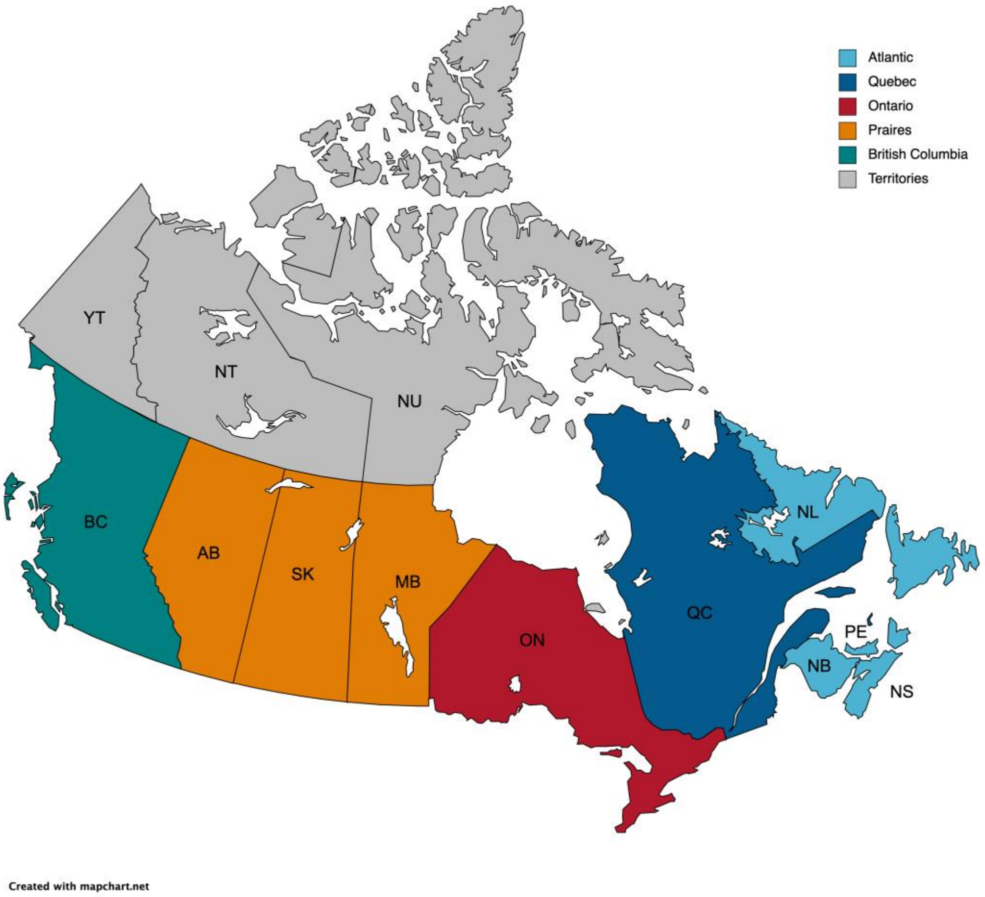

BC: British Columbia, AB: Alberta, SK: Saskatchewan, MB: Manitoba, ON: Ontario, Qc: Quebec, NL: Newfoundland, NB: New Brunswick, PE: Prince Edward Island, NS: Nova Scotia, YT: Yukon Territory, NT Northwest Territory, NU: Nunavut Territory

© 2024 Roumeliotis N et al. *JAMA Pediatr*.

eTable 2. Descriptive characteristics of hospitalizations for eating disorders age 6 to 20 years, by fiscal year in Canada (N=11 289)

	Fiscal Year						
	**2016-17**	**2017-18**	**2018-19**	**2019-20**	**2020-21**	**2021-22**	**2022-23**
Total Hospitalizations N	1200	1192	1298	1294	2082	2347	1876
Age category, n (%)							
6 - 11 years	71 (5.9)	87 (7.3)	93 (7.2)	77 (5.9)	117 (5.6)	120 (5.1)	110 (5.9)
12 - 17 years	985 (82.1)	982 (82.4)	1059 (81.6)	1076 (83.1)	1789 (85.9)	2040 (86.9)	1627 (86.7)
18 - 20 years	144 (12.0)	123 (10.3)	146 (11.2)	141 (10.9)	176 (8.4)	187 (8.0)	139 (7.4)
Sex[a], n (%)							
Female	1092 (91.1)	1067 (89.5)	1157 (89.1)	1163 (89.9)	1909 (91.8)	2160 (92.1)	1663 (88.6)
Male	107 (8.9)	125 (10.5)	141 (10.9)	130 (10.0)	171 (8.2)	185 (7.9)	213 (11.4)
Area[b], n (%)							
Urban	1026 (86.7)	1021 (86.4)	1134 (88.7)	1128 (88.7)	1809 (88.4)	2024 (87.5)	1627 (88.0)
Rural	158 (13.3)	161 (13.6)	144 (11.3)	144 (11.3)	238 (11.6)	290 (12.5)	221 (12.0)
Region, n (%)							
British Columbia	173 (14.4)	177 (14.8)	199 (15.3)	212 (16.4)	317 (15.2)	282 (12.0)	216 (11.5)
Prairies	146 (12.2)	130 (10.9)	153 (11.8)	146 (11.3)	216 (10.4)	268 (11.4)	200 (10.7)
Ontario	493 (41.1)	505 (42.4)	534 (41.1)	551 (42.6)	918 (44.1)	1054 (44.9)	860 (45.8)
Quebec	325 (27.1)	298 (25.0)	330 (25.4)	301 (23.3)	512 (24.6)	612 (26.1)	515 (27.4)
Atlantic	61 (5.1)	82 (6.9)	79 (6.1)	84 (6.5)	115 (5.5)	122 (5.2)	81 (4.3)
Territories	N/R	0 (0.0)	N/R	0 (0.0)	N/R	9 (0.4)	N/R
Pediatric center[c], n (%)							
Yes	666 (55.5)	662 (55.5)	739 (56.9)	761 (58.8)	1245 (59.8)	1295 (55.2)	1023 (54.5)
No	534 (44.5)	530 (44.5)	559 (43.1)	533 (41.2)	837 (40.2)	1052 (44.8)	853 (45.5)
Length of stay days, med (IQR)							
Quebec	28 (10-50)	27 (12-52)	26 (12-47)	25 (10-49)	21 (10-36)	20 (11-42)	23 (11-42)

© 2024 Roumeliotis N et al. *JAMA Pediatr*.

	2016-17	2017-18	2018-19	2019-20	2020-21	2021-22	2022-23
All other provinces	16 (8-35)	17 (8-34)	16 (7-31)	14 (7-30)	14 (7-24)	13 (7-25)	13 (6-22)
Transfer during admission, n (%)	100 (8.3)	105 (8.8)	113 (8.7)	87 (6.7)	171 (8.2)	195 (8.3)	170 (9.1)
Previous Mental Health History in last 2 years, n (%)							
Emergency Department visit	214 (17.8)	217 (18.2)	232 (17.8)	243 (18.8)	390 (18.7)	491 (20.9)	395 (21.1)
Hospitalization	403 (33.6)	381 (32.0)	456 (35.1)	459 (35.5)	602 (28.9)	694 (29.6)	615 (32.8)
None	583 (48.6)	594 (49.8)	610 (47.0)	592 (45.7)	1090 (52.3)	1162 (49.5)	866 (46.1)
Previous Eating Disorder History in last 2 years, n (%)							
Emergency Department visit	116 (9.7)	130 (10.9)	160 (12.3)	134 (10.4)	251 (12.1)	346 (14.7)	261 (13.9)
Hospitalization	377 (31.4)	339 (28.4)	409 (31.5)	404 (31.2)	557 (26.8)	629 (26.8)	561 (29.9)
None	707 (58.9)	723 (60.7)	729 (56.2)	756 (58.4)	1274 (61.2)	1372 (58.5)	1054 (56.2)

N/R: Not reported, Cell count less than 5. Med: median. IQR: Interquartile range

a. N=6 (0.1%) reported as ‘Other’
b. N=164 (1.5%) missing, non-urban refers to rural or remote areas.
c. admission to a pediatric center is defined as a center having a tertiary care pediatric intensive care unit.

© 2024 Roumeliotis N et al. *JAMA Pediatr*.

eTable 3. Canadian population aged 6 to 20 for each fiscal year from 2016 to 2022 stratified by region, sex and age group

Strata	Fiscal year 2016-17	2017-18	2018-19	2019-20	2020-21	2021-22	2022-23
**CANADA**	**6076121**	**6123912**	**6202774**	**6269212**	**6304676**	**6261795**	**6390438**
Male	**3112552**	**3136107**	**3174420**	**3207157**	**3223202**	**3198994**	**3264647**
6-11 years	1209769	1228741	1248146	1262585	1268726	1266731	1281344
12-17 years	1212060	1211455	1213881	1226568	1244756	1256990	1286474
18-20 years	690723	695911	712393	718004	709720	675273	696829
Female	**2963569**	**2987805**	**3028354**	**3062055**	**3081474**	**3062801**	**3125791**
6-11 years	1167196	1184060	1200921	1211683	1214957	1211146	1222797
12-17 years	1153532	1157750	1165306	1180381	1199791	1211903	1241658
18-20 years	642841	645995	662127	669991	666726	639752	661336
**ATLANTIC**	**371143**	**371315**	**372064**	**373681**	**374663**	**371136**	**380438**
Male	**190531**	**190544**	**190541**	**191335**	**191835**	**190046**	**194874**
6-11 years	72554	73143	73494	73614	73492	73136	74127
12-17 years	75590	74970	74180	74694	75479	76052	78319
18-20 years	42387	42431	42867	43027	42864	40858	42428
Female	**180612**	**180771**	**181523**	**182346**	**182828**	**181090**	**185564**
6-11 years	69117	69833	70310	70523	70153	69623	70462
12-17 years	72195	71774	71486	71803	72708	72828	74956
18-20 years	39300	39164	39727	40020	39967	38639	40146
**QUEBEC**	**1287873**	**1296080**	**1313282**	**1335353**	**1356037**	**1357072**	**1375050**
Male	**657425**	**661613**	**669798**	**681648**	**692394**	**692376**	**701118**
6-11 years	265013	272364	279712	285309	288107	286582	286997
12-17 years	249068	249046	251467	256716	263362	269842	277749
18-20 years	143344	140203	138619	139623	140925	135952	136372
Female	**630448**	**634467**	**643484**	**653705**	**663643**	**664696**	**673932**
6-11 years	255700	262480	269236	273928	276527	275127	274788
12-17 years	238304	238718	241699	247216	253797	260305	268208
18-20 years	136444	133269	132549	132561	133319	129264	130936
**ONTARIO**	**2402088**	**2417569**	**2447055**	**2462779**	**2463205**	**2427970**	**2473364**
Male	**1230055**	**1238571**	**1253947**	**1260674**	**1259568**	**1240538**	**1264729**
6-11 years	465301	468169	472137	474329	473904	472380	477648
12-17 years	484245	484370	484198	486832	490526	490505	496792
18-20 years	280509	286032	297612	299513	295138	277653	290289
Female	**1172033**	**1178998**	**1193108**	**1202105**	**1203637**	**1187432**	**1208635**
6-11 years	449586	451747	455323	456864	455941	453832	457626
12-17 years	461074	462563	464126	467408	471293	471419	478197
18-20 years	261373	264688	273659	277833	276403	262181	272812
**PRAIRIES**	**1228499**	**1245378**	**1265485**	**1285536**	**1299256**	**1303823**	**1336771**
Male	**629454**	**637719**	**647833**	**658195**	**664837**	**666637**	**683019**
6-11 years	256786	262983	268456	272752	274926	275216	279841

© 2024 Roumeliotis N et al. *JAMA Pediatr*.

12-17 years	243480	244464	246327	250280	256084	261157	269501
18-20 years	129188	130272	133050	135163	133827	130264	133677
Female	**599045**	**607659**	**617652**	**627341**	**634419**	**637186**	**653752**
6-11 years	247975	253557	257904	260944	262334	262400	267108
12-17 years	231822	233576	236263	241292	247636	252549	260408
18-20 years	119248	120526	123485	125105	124449	122237	126236
**BRITISH COLUMBIA**	**761074**	**767814**	**778780**	**785448**	**784535**	**774495**	**797195**
Male	**392093**	**394594**	**399092**	**402000**	**401000**	**395653**	**406988**
6-11 years	144599	146456	148615	150769	152389	153412	156716
12-17 years	154801	153680	152777	153030	154109	154102	158650
18-20 years	92693	94458	97700	98201	94502	88139	91622
Female	**368981**	**373220**	**379688**	**383448**	**383535**	**378842**	**390207**
6-11 years	139547	141010	142609	143762	144241	144315	147034
12-17 years	145329	146197	146786	147638	149213	149550	154484
18-20 years	84105	86013	90293	92048	90081	84977	88689
**TERRITORIES**	**25444**	**25756**	**26108**	**26415**	**26980**	**27299**	**27620**
Male	**12994**	**13066**	**13209**	**13305**	**13568**	**13744**	**13919**
6-11 years	5516	5626	5732	5812	5908	6005	6015
12-17 years	4876	4925	4932	5016	5196	5332	5463
18-20 years	2602	2515	2545	2477	2464	2407	2441
Female	**12450**	**12690**	**12899**	**13110**	**13412**	**13555**	**13701**
6-11 years	5271	5433	5539	5662	5761	5849	5779
12-17 years	4808	4922	4946	5024	5144	5252	5405
18-20 years	2371	2335	2414	2424	2507	2454	2517

© 2024 Roumeliotis N et al. *JAMA Pediatr*.

eTable 4. Unadjusted incidence rate ratios (IRR) for eating disorders, comparing hospitalization rates in pre-COVID and COVID-prevalent years, stratified by region, sex and age.

Region	Pre-COVID Rate per 100 000	IRR (95%CI) 2020 – 2021	IRR (95%CI) 2021 – 2022	IRR (95%CI) 2022 – 2023
**CANADA**	20.19	1.63 (1.55-1.72)	1.85 (1.75-1.95)	1.45 (1.38-1.53)
Male	3.98	1.33 (1.11-1.59)	1.45 (1.22-1.72)	1.64 (1.39-1.93)
Female	37.20	1.67 (1.58-1.76)	1.90 (1.80-2.00)	1.43 (1.35-1.51)
6-11 years	4.87	1.52 (1.18-1.95)	1.56 (1.21-1.99)	1.41 (1.09-1.82)
12-17 years	80.10	1.72 (1.62-1.82)	1.95 (1.84-2.06)	1.46 (1.37-1.55)
18-20 years	19.73	1.26 (1.05-1.51)	1.40 (1.18-1.67)	0.97 (0.80-1.18)
**ATLANTIC**	20.56	1.49 (1.19-1.86)	1.60 (1.29-1.98)	1.04 (0.80-1.33)
Male	3.41	1.07 (0.39-2.53)	1.70 (0.76-3.56)	1.51 (0.65-3.23)
Female	38.61	1.53 (1.21-1.92)	1.59 (1.26-1.98)	0.99 (0.72-1.29)
6-11 years	--	--	--	--
12-17 years	74.50	1.50 (1.14-1.94)	1.51 (1.16-1.96)	0.91 (0.66-1.24)
18-20 years	32.24	1.79 (1.04-2.97)	2.17 (1.31-3.52)	1.08 (0.55-1.98)
**QUEBEC**	23.97	1.58 (1.42-1.75)	1.88 (1.71-2.07)	1.56 (1.41-1.73)
Male	3.82	1.40 (0.93-2.06)	1.25 (0.82-1.86)	1.98 (1.39-2.78)
Female	44.96	1.59 (1.43-1.77)	1.94 (1.75-2.14)	1.52 (1.37-1.70)
6-11 years	4.05	2.59 (1.56-4.24)	2.51 (1.50-4.14)	1.98 (1.13-3.38)
12-17 years	101.66	1.57 (1.39-1.76)	1.92 (1.72-2.14)	1.50 (1.33-1.69)
18-20 years	23.75	1.33 (0.91-1.89)	1.37 (0.94-1.95)	1.00 (0.65-1.49)
**ONTARIO**	21.41	1.74 (1.61-1.88)	2.03 (1.88-2.18)	1.62 (1.50-1.76)
Male	5.24	1.27 (0.98-1.63)	1.59 (1.25-2.00)	1.66 (1.32-2.08)
Female	38.37	1.80 (1.66-1.96)	2.08 (1.92-2.25)	1.62 (1.48-1.76)
6-11 years	6.73	1.08 (0.71-1.59)	1.21 (0.82-1.76)	1.20 (0.81-1.75)
12-17 years	88.67	1.86 (1.71-2.03)	2.12 (1.95-2.30)	1.62 (1.48-1.78)
18-20 years	5.01	1.44 (0.82-2.45)	1.98 (1.19-3.22)	1.76 (1.04-2.89)
**PRAIRIES**	11.44	1.45 (1.24-1.70)	1.80 (1.55-2.08)	1.31 (1.11-1.54)
Male	1.71	0.70 (0.29-1.51)	1.75 (0.98-3.04)	2.05 (1.20-3.45)
Female	21.66	1.51 (1.28-1.78)	1.80 (1.54-2.09)	1.24 (1.04-1.48)
6-11 years	2.65	2.16 (1.07-4.21)	2.02 (0.98-3.98)	1.41 (0.61-3.02)
12-17 years	36.91	1.77 (1.46-2.14)	2.15 (1.79-2.56)	1.50 (1.23-1.82)
18-20 years	31.94	0.78 (0.51-1.15)	0.87 (0.58-1.27)	0.55 (0.33-0.86)
**BRITISH COLUMBIA**	24.60	1.64 (1.44-1.87)	1.48 (1.29-1.70)	1.10 (0.94-1.28)
Male	4.41	1.98 (1.28-3.01)	1.03 (0.58-1.75)	0.89 (0.48-1.55)
Female	45.84	1.60 (1.39-1.85)	1.52 (1.31-1.75)	1.12 (0.95-1.31)
6-11 years	4.41	1.41 (0.58-3.13)	1.73 (0.77-3.64)	1.39 (0.57-3.07)
12-17 years	91.48	1.63 (1.39-1.91)	1.51 (1.28-1.77)	1.10 (0.91-1.31)
18-20 years	36.60	1.52 (1.07-2.12)	1.51 (1.06-2.13)	1.11 (0.74-1.62)
**TERRITORIES**	--	--	--	--

IRR: Incidence Rate Ratio. N/R: Not reported as counts too low.

© 2024 Roumeliotis N et al. *JAMA Pediatr*.

Fiscal years are April 1st to March 31st.
Males not stratified by age groups as counts too low.
Territories not reported as sex stratified counts per year too low.

© 2024 Roumeliotis N et al. *JAMA Pediatr*.

eFigure 3. Interrupted time series of fitted vs. expected rates of eating disorder hospitalizations among females 12 to 17 years of age, by region and 4-week period, from pre-pandemic trends

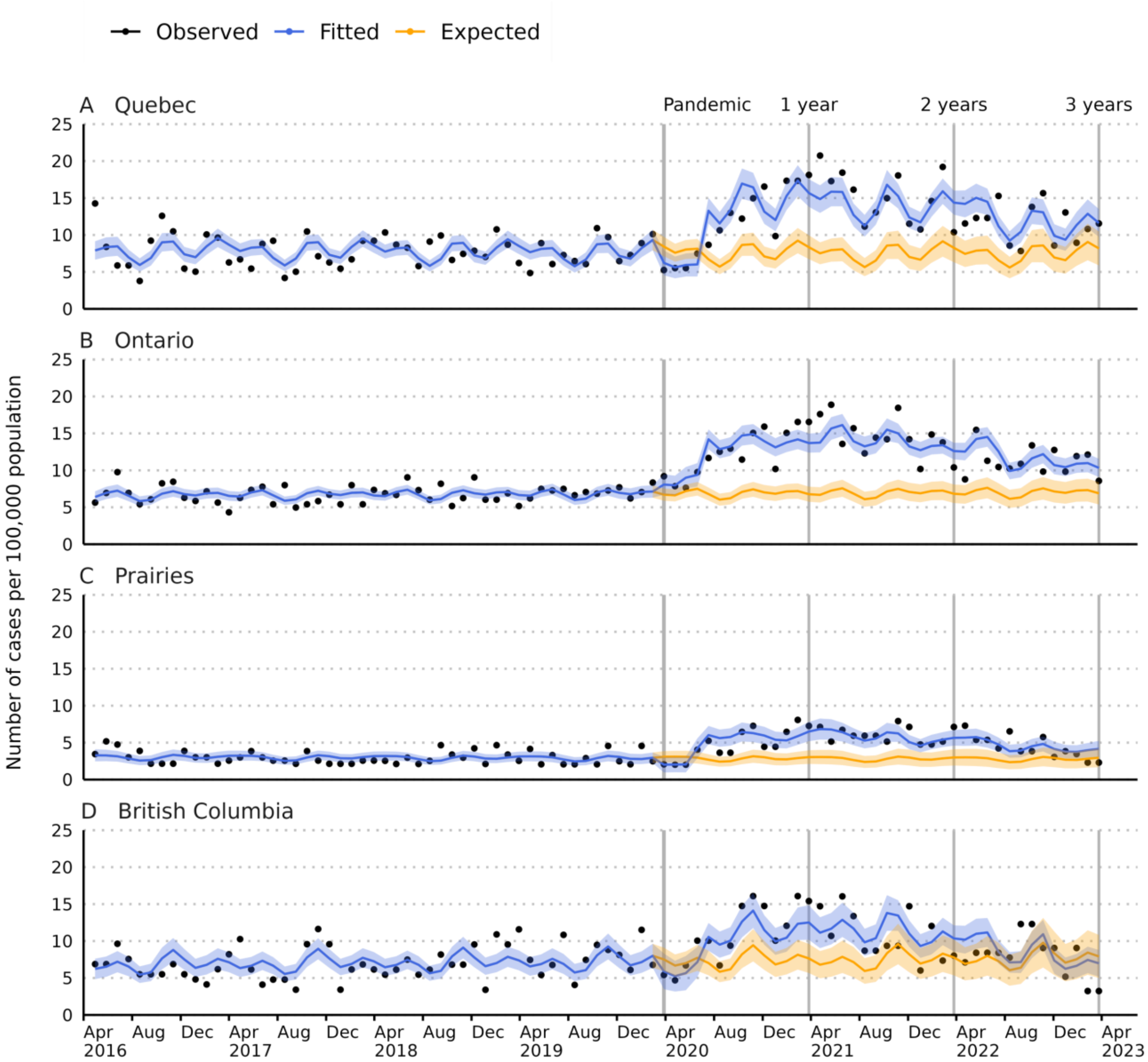

The shaded areas represent the 95% confidence interval for the fitted curve (blue) and the expected curve (orange).
Series runs from April 2016 to March 2023.

© 2024 Roumeliotis N et al. *JAMA Pediatr*.

eTable 5. Change in volume of eating disorder hospitalizations among females 12 to 17 years, comparing the pre-pandemic trend from the regression model extrapolated into the COVID-prevalent period and the fitted rate.

**Region**<br>Time since pandemic	**Expected rate per 100,000 population**	**Fitted rate per 100,000 population**	**Rate ratio**
**Quebec**			
1 year	8.34	15.68	1.88
2 years	8.26	14.38	1.74
3 years	8.18	11.62	1.42
**Ontario**			
1 year	6.77	13.69	2.02
2 years	6.83	12.61	1.85
3 years	6.89	10.33	1.50
**Prairies**			
1 year	3.04	6.50	2.14
2 years	3.00	5.65	1.88
3 years	2.96	4.16	1.41
**British Columbia**			
1 year	7.65	12.51	1.64
2 years	7.78	10.36	1.33
3 years	7.92	6.99	0.88

Pandemic start is April 1st, 2020.

© 2024 Roumeliotis N et al. *JAMA Pediatr*.

eTable 6. Sensitivity analysis, varying the lag of the stringency and the duration of the healthcare restriction.

Model parameters		BG test p-value			BIC	Exp(coefficient) for the stringency index (95%CI)
Healthcare restriction period	Stringency lag	Order 1	Order 2	Order 3		
**Quebec**						
12 weeks	12 weeks	0.1037	0.2108	0.2238	622.74	1.04 (1.01-1.06) *
	16 weeks	0.1257	0.2111	0.2438	611.45	1.06 (1.03-1.08) ***
	20 weeks	0.4182	0.6746	0.6144	607.04	1.06 (1.03-1.09) ***
16 weeks	12 weeks	0.2916	0.3909	0.4583	609.75	1.04 (1.01-1.07) **
	16 weeks	0.3400	0.4568	0.5188	609.77	1.05 (1.01-1.07) **
	20 weeks	0.4401	0.6339	0.6528	605.69	1.05 (1.02-1.08) ***
**Ontario**						
12 weeks	12 weeks	0.3779	0.6742	0.4014	661.70	1.05 (1.03-1.07) ***
	16 weeks	0.7546	0.9063	0.2620	656.02	1.05 (1.03-1.07) ***
	20 weeks	0.7574	0.9380	0.3639	657.30	1.05 (1.03-1.07) ***
16 weeks	12 weeks	0.6869	0.9205	0.3910	652.31	1.05 (1.03-1.06) ***
	16 weeks	0.9841	0.9681	0.2153	654.96	1.05 (1.03-1.07) ***
	20 weeks	0.7598	0.9349	0.2886	655.44	1.04 (1.02-1.06) ***
**Prairies**						
12 weeks	12 weeks	0.1353	0.3257	0.3269	474.09	1.07 (1.01-1.13)
	16 weeks	0.1830	0.3357	0.4728	471.27	1.08 (1.03, 1.13) *
	20 weeks	0.4571	0.6352	0.6991	468.95	1.08 (1.03-1.14) **
16 weeks	12 weeks	0.1906	0.3178	0.4516	471.64	1.08 (1.03-1.13) *
	16 weeks	0.2552	0.3007	0.4827	473.92	1.07 (1.02-1.13)
	20 weeks	0.3217	0.2919	0.4660	471.47	1.08 (1.03-1.14) *
**British Columbia**						
12 weeks	12 weeks	0.0500	0.0675	0.0745	520.58	1.13 (1.07-1.19) ***
	16 weeks	0.0719	0.1297	0.1499	527.21	1.11 (1.05-1.16) **
	20 weeks	0.0420	0.0956	0.1532	529.91	1.09 (1.04-1.14)
16 weeks	12 weeks	0.0540	0.0754	0.0825	520.49	1.13 (1.08-1.18) ***
	16 weeks	0.0778	0.1460	0.1545	527.01	1.12 (1.07-1.18) **
	20 weeks	0.0472	0.1228	0.1749	530.47	1.11 (1.05-1.16) *

BG: Breusch-Godfrey test for higher-order serial correlation. BIC: Bayesian Information Criterion.
CI: Confidence Interval.
Highlighted in green: the final model selected. P-value * <0.05 ** <0.01 *** <0.001

© 2024 Roumeliotis N et al. *JAMA Pediatr*.
