# Supplementary material for: Pandemic Stringency Measures and Hospital Admissions for Eating Disorders
Source: JAMA Pediatr. 2024 Jul 8;178(9):879–87. doi: 10.1001/jamapediatrics.2024.2044 (PMC11231909; doi:10.1001/jamapediatrics.2024.2044)
Supplement: Supplement 2. — Pediatric Outcomes Improvement Through Coordination of Research Networks (POPCORN) Investigators [file jamapediatr-e242044-s002.pdf]

*Group Name(s): POPCORN Investigators							
***First Name and Middle Initial(s)**	***Last Name**	***Suffix (eg, Jr, III)**	Academic Degrees	Institution	Location (city, state/province, country)	Role or Contribution, eg, chair, principal investigator	Group (if more than 1 Group listed in the byline) and/or Subgroup (eg, Steering Committee)
Sarah	Ahira			Maternal Infant Child Youth Research Network	Calgary, AB, Canada	MICYRN Clinical Research Project Manager/POPCORN Platform Manager	
Samina	Ali			University of Alberta	Edmonton, AB, Canada	Indirect Consequences Project team member / Co-A UofA	
Upton	Allen			University of Toronto	Toronto, ON, Canada	EDI + I team member / Co-A SickKids	
Krista	Baerg			University of Saskatchewan	Saskatoon, SK, Canada	PIRN site lead	
Megan	Bale-Nick			University of Manitoba	Winnipeg, MN, Canada	Knowledge Broker	
Ananya	Banerjee			McGill University	Montreal, QC, Canada	EDI + I team member / Co-A McGill	
Michelle	Barton			Western University	London, ON, Canada	ID/IMPACT site lead	
Darcy	Beer			University of Manitoba	Winnipeg, MN, Canada	PERC site lead / POPCORN Site PI - CHRIM	
Simon	Berthelot			Laval University	Quebec city, QC, Canada	PERC site lead	
Julie	Bettinger			University of British Columbia	Vancouver, BC, Canada	Epid/ Biostats pillar team member	
Maala	Bhatt			University of Ottawa	Ottawa, ON, Canada	POPCORN & CURNLS site PI - CHEO	
Melanie	Buba			University of Ottawa	Ottawa, ON, Canada	PIRN site lead	
Francine	Buchanan			University of Toronto	Toronto, ON, Canada	Patient engagement lead	
Jared	Bullard			University of Manitoba	Winnipeg, MN, Canada	ID/IMPACT site lead	
Brett	Burstein			McGill University	Montreal, QC, Canada	Acute COVID Project Lead / Scientific committee member	
Catherine	Burton			University of Alberta	Edmonton, AB, Canada	Vaccine Safety Project team member / Co-A UofA	
Rahul	Chanchlani			McMaster University	Hamilton, ON, Canada	Longterm Outcomes Project team member / Co-A McMasters	
Michaël	Chassé			University of Montreal	Montreal, QC, Canada	Data governance team member	
Karen	Choong			McMaster University	Hamilton, ON, Canada	Longterm Outcomes Project Lead / Scientific committee member	

***First Name and Middle Initial(s)**	***Last Name**	***Suffix (eg, Jr, III)**	Academic Degrees	Institution	Location (city, state/province, country)	Role or Contribution, eg, chair, principal investigator	Group (if more than 1 Group listed in the byline) and/or Subgroup (eg, Steering Committee)
Evelyn	Constantin			McGill University	Montreal, QC, Canada	Mentorship and Traning Director / Longterm Outcomes Project team member / PA RI-MUHC	
Cora	Constantinescu			University of Calgary	Calgary, AB, Canada	ID/IMPACT site lead	
Carrie	Costello			Children's Hospital Research Institute of Manitoba	Winnipeg, MN, Canada	Former PE Co-Lead	
Sylvana	Côté			University of Montreal	Montreal, QC, Canada	Education/ social science expert - OPES	
Tammie	Dewan			University of Calgary	Calgary, AB, Canada	PIRN site lead	
Tanya	Di Genova			McGill University	Montreal, QC, Canada	Indirect Consequences Project team member / Co-A McGill	
Olivier	Drouin			University of Montreal	Montreal, QC, Canada	Vaccine Safety Project team member and ECR/KM / POPCORN Site PI - CHUSJ / Knowledge mobilization Pillar lead	
Karen	Dryden-Palmer			University of Toronto	Toronto, ON, Canada	Data governance team member	
Geneviève	Du Pont-Thibodeau			University of Montreal	Montreal, QC, Canada	Longterm outcomes Co-project Lead / Co-A CHUSJ	
Marc-André	Dugas			Laval University	Quebec city, QC, Canada	POPCORN & CURNLS site PI - CHUL / East Site Rep Scientific committee	
Raven	Dumont-Maurice			McGill University	Montreal, QC, Canada	EDI + I team member / Collab Montreal Children's Hospital	
Guillaume	Emeriaud			University of Montreal	Montreal, QC, Canada	Acute COVID Project member / Co-A CHUSJ	
Jason	Emsley			Dalhousie University	Halifax, NS, Canada	PERC site lead	
Mark	Ferro			University of Waterloo	Waterloo, ON, Canada	Longterm Outcomes Project team member / Co-A UofWaterloo	
Karen	Forbes			University of Montreal	Montreal, QC, Canada	Acute COVID Project member / Co-A UofA	
Isabel	Fortier			McGill University	Montreal, QC, Canada	Data governance team member	
Jennifer	Foster			Dalhousie University	Halifax, NS, Canada	POPCORN site PI - IWK	
Jessica	Foulds			University of Alberta	Edmonton, AB, Canada	Longterm Outcomes Project team member / Co-A UofA	

***First Name and Middle Initial(s)**	***Last Name**	***Suffix (eg, Jr, III)**	Academic Degrees	Institution	Location (city, state/province, country)	Role or Contribution, eg, chair, principal investigator	Group (if more than 1 Group listed in the byline) and/or Subgroup (eg, Steering Committee)
Stephen	Freedman			University of Calgary	Calgary, AB, Canada	Emergency medicine Pillar lead / POPCORN & CURNLS site PI - ACH / Scientific committee member	
Gabrielle	Freire			University of Toronto	Toronto, ON, Canada	Longterm Outcomes Project team member / Co-A Sickkids	
Eleni	Galanis			Public Health Agency of Canada	Ottawa, ON, Canada	Principal knowledge user Co-chair / PHAC	
Peter	Gill			University of Toronto	Toronto, ON, Canada	Researcher and ECR rep; Acute COVID Project member/PA Sickkids / POPCORN ECR on Scientific committee	
Jocelyn	Gravel			University of Montreal	Montreal, QC, Canada	Acute COVID Project member / Co-A CHUSJ	
Emily	Gruenwoldt			Children's Healthcare Canada	Ottawa, ON, Canada	Knowledge User	
Gonzalo	Guerra			University of Alberta	Edmonton, AB, Canada	Longterm Outcomes Project team member / Collab. UofA	
Astrid	Guttman			Institute for Clinical Evaluative Sciences	Toronto, ON, Canada	Longterm Outcomes Project team member / Collab. UofT	
Betty Jean	Hancock			Health Sciences Centre Winnipeg	Winnipeg, MN, Canada	CCCTG site lead	
Robyn	Harrison			University of Alberta	Edmonton, AB, Canada	Knowledge User - UofC	
Joanna	Holland			Dalhousie University	Halifax, NS, Canada	PIRN site lead	
Ari	Joffe			University of Alberta	Edmonton, AB, Canada	Acute COVID Project member / Co-A Stollery	
Fatima	Kakkar			University of Montreal	Montreal, QC, Canada	Former site PI	
April	Kam			McMaster University	Hamilton, ON, Canada	PERC site lead	
James D.	Kellner			University of Calgary	Calgary, AB, Canada	Scientific committee chair	
Lisa	Knisley			Children's Hospital Research Institute of Manitoba	Winnipeg, MN, Canada	Knowledge mobilization team member / co-A TREKK / CHRIM / POPCORN Early Career Researcher (ENRICH)	
Thierry	Lacaze-Masmonteil			Maternal Infant Child Youth Research Network	Ottawa, ON, Canada	Coordinating center lead/ MICYRN	

***First Name and Middle Initial(s)**	***Last Name**	***Suffix (eg, Jr, III)**	Academic Degrees	Institution	Location (city, state/province, country)	Role or Contribution, eg, chair, principal investigator	Group (if more than 1 Group listed in the byline) and/or Subgroup (eg, Steering Committee)
Saptharishi	Lalgudi Ganesan			University of Western Ontario	London, ON, Canada	Data governance team member / POPCORN & CURNLS site PI - LHSC / Central Site Rep Scientific committee	
Marc- André	Langlois			University of Ottawa	Ottawa, ON, Canada	Biobank governance team member	
Nicole	Le Saux			University of Ottawa	Ottawa, ON, Canada	ID/IMPACT site lead	
Laurie	Lee			University of Calgary	Calgary, AB, Canada	CCCTG site lead	
Kirk	Leifso			Queen's University	Kinston, ON, Canada	ID/IMPACT site lead	
Patricia	Li			McGill University	Montreal, QC, Canada	EDI + I director / Scientific committee member	
Andrea	Linares			Maternal Infant Child Youth Research Network	Calgary, AB, Canada	MICYRN Clinical Research Project Manager	
Sanjay	Mahant			University of Toronto	Toronto, ON, Canada	Inpatient medicine Pillar lead / Scientific committee member	
Isabelle	Marc			Laval University	Quebec city, QC, Canada	PIRN site lead	
Ahmed	Mater			University of Saskatchewan	Saskatoon, SK, Canada	POPCORN & CURNLS site PI - JPCH	
James	McNally			University of Ottawa	Ottawa, ON, Canada	Knowledge mobilization team member / PICU Rep	
Garth	Meckler			University of British Columbia	Vancouver, BC, Canada	Longterm Outcomes Project team member / Co-A UBC	
Shaun	Morris			University of Toronto	Toronto, ON, Canada	Acute COVID Project team member and POPCORN site PI - Sickkids	
Haifa	Mtaweh			University of Toronto	Toronto, ON, Canada	CCCTG site lead	
Srin	Murthy			University of British Columbia	Vancouver, BC, Canada	Data governance team member / Governance Committee Co-chair	
Fiona	Muttalib			University of British Columbia	Vancouver, BC, Canada	Vaccine Safety Project team member / CURNLS site PA - CWBC / Western Site Rep Scientific committee	
Leigh- Anne	Newhook			Memorial University	St-John's, NL, Canada	PIRN site lead	
Jessica	Nicoll			Memorial University	St-John's, NL, Canada	CCCTG site lead	

*First Name and Middle Initial(s)	*Last Name	*Suffix (eg, Jr, III)	Academic Degrees	Institution	Location (city, state/province, country)	Role or Contribution, eg, chair, principal investigator	Group (if more than 1 Group listed in the byline) and/or Subgroup (eg, Steering Committee)
Nathalie	Orr-Gaucher			University of Montreal	Montreal, QC, Canada	Vaccine Safety Project team member	
Joseph	Pagano			University of Alberta	Edmonton, AB, Canada	Vaccine Safety Project team member / Co-A Stollery	
Anna	Pangilinan			Maternal Infant Child Youth Research Network	Vancouver, BC, Canada	MICYRN Administrative Assistant	
Jesse	Papenburg			McGill University	Montreal, QC, Canada	Indirect Consequences Project team member and POPCORN site PI - HME / IMPACT Rep - HME	
Jeffrey	Pernica			McGill University	Montreal, QC, Canada	ID/IMPACT site lead	
Naveen	Poonai			University of Western Ontario	London, ON, Canada	Vaccine Safety Project team member / Co-A Western	
Elodie	Portales-Casamar			University of Montreal	Montreal, QC, Canada	Data governance co-lead / Scientific committee member	
Robert	Porter			Memorial University	St-John's, NL, Canada	PERC site lead	
Rupeena	Purewal			University of Saskatchewan	Saskatoon, SK, Canada	ID/IMPACT site lead	
Paula	Robeson			Children's Healthcare Canada	Ottawa, ON, Canada	Knowledge User/KM team	
Joan	Robinson			University of Alberta	Edmonton, AB, Canada	Mentorship and training team member / Co-A UofA	
Manish	Sadarangani			University of British Columbia	Vancouver, BC, Canada	Infectious diseases Pillar lead / Scientific committee member	
Marina	Salvadori			McGill University	Montreal, QC, Canada	PHAC rep on Scientific committee/ Knowledge user / Co-A PHAC	
Susan	Samuel			University of Calgary	Calgary, AB, Canada	Mentorship and training team member / Collab. UofC	
Shannon	Scott			University of Alberta	Edmonton, AB, Canada	KM Expert - ARCHE	
Anupam	Sehgal			Queen's University	Kingston, ON, Canada	POPCORN & CURNLS site PI - KHSC	
Archna	Shah			Memorial University	St-John's, NL, Canada	POPCORN & CURNLS Site PI - JCH	
Tatiana	Sotindjo			University of British Columbia	Vancouver, BC, Canada	EDI + I team member / Co-A UBC	
Carla	Southward			University of Toronto	Toronto, ON, Canada	Patient engagement team member / Patient Engagement Coordinator - SickKids	
Taylor	Stoesz			McGill University	Montreal, QC, Canada	EDI + I team member / Co-A McGill	

***First Name and Middle Initial(s)**	***Last Name**	***Suffix (eg, Jr, III)**	Academic Degrees	Institution	Location (city, state/province, country)	Role or Contribution, eg, chair, principal investigator	Group (if more than 1 Group listed in the byline) and/or Subgroup (eg, Steering Committee)
Robert	Strang			Chief Medical Officers Health - Department of Health and Wellness	Halifax, NS, Canada	Principal knowledge user	
Shazeen	Suleman			St. Michael's Hospital Toronto	Toronto, ON, Canada	EDI + I team member / Co-A St. Michael's Hospital Toronto	
Peter	Szatmari			University of Toronto	Toronto, ON, Canada	Mental Health expert	
Sepi	Taheri			University of Western Ontario	London, ON, Canada	PIRN site lead	
Jennifer	Tam			University of British Columbia	Vancouver, BC, Canada	Mentorship and training team member / co-A UBC	
Roseline	Thibeault			Laval University	Quebec city, QC, Canada	ID/IMPACT site lead	
Karina	Top			Dalhousie University	Halifax, NS, Canada	Vaccine Safety Project lead / Scientific committee member	
Krystel	Toulouse			Université de Sherbrooke	Sherbrooke, QC, Canada	POPCORN site PI - CHUSB	
Sze-Man	Tse			University of Montreal	Montreal, QC, Canada	Biobank governance team member	
Anupma	Wadhwa			University of Toronto	Toronto, ON, Canada	Longterm Outcomes Project team member	
Gita	Wahi			McMaster University	Hamilton, ON, Canada	Indirect Consequences Project team member and POPCORN & CURNLS site PA - MCH	
Sam	Wong			University of Alberta	Edmonton, AB, Canada	EDI + I team member / Co-A UofA	
Bruce	Wright			University of Alberta	Edmonton, AB, Canada	POPCORN & CURNLS site PI - SCH	
Rae	Yeung			University of Toronto	Toronto, ON, Canada	Vaccine Safety Project team member / Co-A Sickkids / Biobank governance team member	
