# Supplementary material for: Pandemic Stringency Measures and Hospital Admissions for Eating Disorders
Source: JAMA Pediatr. 2024 Jul 8;178(9):879–87. doi: 10.1001/jamapediatrics.2024.2044 (PMC11231909; doi:10.1001/jamapediatrics.2024.2044)
Supplement: Supplement 3. — Data Sharing Statement [file jamapediatr-e242044-s003.pdf]

Data Sharing Statement

Roumeliotis. Pandemic Stringency Measures and Hospital Admissions for Eating Disorders. *JAMA Pediatr*. Published July 08, 2024. doi:10.1001/jamapediatrics.2024.2044

Data
Data available: No

Additional Information
Explanation for why data not available: Health administrative data for this study was provided by the Canadian Institut for Health Information (CIHI) under strict access in a secure environment. Individual record level data sharing is not permitted by CIHI, however aggregate data can be made available upon request to primary authors, with agreements from POPCORN network and CIHI respected.
